# Cannabis Use in Muslim Youth

**DOI:** 10.7759/cureus.15615

**Published:** 2021-06-12

**Authors:** Reyam N Nassif

**Affiliations:** 1 Health Sciences, McMaster University, Hamilton, CAN

**Keywords:** cannabis, religions, islam, legalization, youth

## Abstract

Cannabis is the most used illicit drug in the world. It causes impaired executive functioning, psychosis, and schizophrenia, among other impairments. It also affects reaction time, awareness, and motivation. These side effects can lead to decreased academic performance as well as social setbacks. Variance in the interpretation of whether cannabis is forbidden fuels the ongoing debate on the religious stance of cannabis use among Muslim communities across the globe. Stigma is the biggest barrier for open discussion about cannabis usage and also acts as a barrier to the implementation of harm-reductive programs in the Islamic world. There is clear evidence that due to stigma, religious beliefs, and social factors, Muslim youth are at a higher risk than their adult counterparts and that they feel unable to seek help with regard to cannabis and other drug abuse. By reviewing studies on the harmful effects of cannabis use and comparing them against notions of what is considered forbidden in the Islamic tradition and other communities, this paper explores the best ways to reduce harm from cannabis usage in the global Muslim community.

## Introduction and background

Islam is a religion that serves and prescribes a way of life for Muslims in a protective and unifying way [1]. Islam has been embraced by many societies and ethnic groups, and its followers’ diversity makes the understanding of Islam a complex task. The cannabis plant is the most widely used and vastly grown drug in many parts of the world [2]. It grows naturally in the wild and is also cultivated [2]. Research has found that teenagers and adolescents are most likely to engage in cannabis use. However, there is a decline in usage into early and late adulthood [3]. Tetrahydrocannabinol (THC), the psychoactive component in cannabis, binds to cannabinoid receptors in the central nervous, respiratory, system, and urinary systems [3,4]. Cannabis’s effect on the body through these systems influences appetite, sexual activity, mood, nausea, and pain [3]. The risk of psychiatric disorders encompasses psychotic disorders, including schizophrenia. Longitudinal data indicates an association between adolescent cannabis use and psychosis [5]. In adolescents, research has found a neuropsychological decline in cannabis users aged 13 years and older. Structural changes in the medial temporal amygdala, hippocampus, frontal, and cerebral regions can also occur due to cannabis use [5]. When researching adolescent cannabis use, it has been found that extensive use can affect brain development by altering its circuitry [3].

Cannabis use does come with further consequences. Cannabis intoxication includes a euphoric feeling, laugher, increase in appetite, restlessness, forgetfulness, dry mouth, increased heart rate, and reddened eyes [3]. Although illicit drug use is considered forbidden, many people in the Islamic world engage in such activities [6]. When considering the use of cannabis, there are also considerable religious and philosophical sentiments within the global Islamic community. These debates regarding the forbidden use of cannabis go back many years and have been studied by scientists, scholars, poets, and historians [7]. Cannabis was permissible in Islam even before it was used for medicinal purposes in Iran. Islam did not prohibit the use of illicit drugs and alcohol until years after the revelation established the basics of the religion. It was not until cannabis became associated with war, violence, and malevolent behavior that it became forbidden in Islam. However, the debates addressing whether or not its use should be permissible are relevant to today’s theologians and philosophers of Islam.

Muslim countries are beginning to see an increase in the use of cannabis [7]. In Egypt, cannabis addiction is on the rise and is creating dangerous habits, including driving while under its influence [8]. It poses a threat to road and traffic safety in Egypt as cannabis makes the driver significantly less aware and cognizant of their surroundings. A study has indicated that driving under the effect of cannabis is similar to driving under the effect of alcohol, which results in negative effects not only on the consumer but also upon society [8].

## Review

Legislation

It has been said that the permissible use or prohibition of cannabis is based on the similar concept of forbidding alcohol [9]. With this in mind, a Muslim may ponder the ambiguity of cannabis as it does not fully fit the Islamic definition of an intoxicating substance. Such substances are prevalent in Islamic countries, especially among university students [10]. Among drug users, tobacco use is also prevalent [10].

Any behavior that may cause an individual to act in a malevolent way, causing harm to either oneself or society, is forbidden in Islam in order to lower the corruption rate among society [10]. Kamarulzaman and Saifuddeen refer to this as harm reduction, which is the purpose of Islamic law [6]. According to the Islamic religion, five aspects of life are to be protected: faith, life, intellect, progeny, and wealth. These are noted as critical in maintaining and reproducing healthy human life and relationships [6]. Because harm reduction is of high importance in Islam, introducing harm reduction strategies for Muslim youth should be of equally high importance. Harm reduction is meant to reduce any distress that can be inflicted upon a person by themselves or others, leading to a more joyful and peaceful life. Programs aimed at harm reduction, such as dafu al-dharar wa jalbul manfaat or harm must be treated and benefits must be brought forth, have proven to be very successful in achieving these goals [6]. Furthermore, the focus of Islam is not solely on preventing and reducing anguish but is about giving a more abundant life - especially in relation to health [11]. Sattari et al. argued that the use of any narcotic material including cannabis will lead to the same disastrous and harmful behaviors as alcohol [10]. Currently, cannabis is consumed solely, but over time there is a high chance that the user will seek more potent and intoxicating drugs that offer more intense physical and mental alterations [10].

Cannabis influence

According to Chekib et al., there are several consequences of cannabis consumption [12]. It increases rates of early death, disability, and chronic disease as well as has a great impact on the social, cultural, and economic status of individuals and the community. It can lead to depression, homicides, suicides, violence, injuries, and accidents. It has also been associated with academic underperformance and unwanted sexual encounters. In Tunisia, a study monitoring the prevalence rate of illicit drug use and its predictors in college students using a self-administrated questionnaire found that almost 5% of the participants were using cannabis and experiencing significant academic failure [12]. In Saudi Arabia, using cannabis while driving is equivalent to driving under the influence of alcohol [13]. Driving under the influence of cannabis is linked to other destructive behaviors including road rage and carrying weapons [13].

Age and prevalence

In Turkey, Telo et al. examined the prevalence of drug use by screening the urine of patients who visited Elazig Mental Hospital, Elazığ, Turkey [14]. In their retrospective study, they found that cannabis was the most common drug used by young adults between the ages of 20 and 29 years [14]. According to the United Nations Office on Drugs and Crime 2010, cannabis is the most consumed illicit drug with an estimated 129-190 million users globally [15]. Bassiony reviewed 21 studies that examined substance abuse in Saudi Arabia. The outcomes revealed that most substance abuse among Saudis in the last decade was via cannabis (60%) and amphetamines (4%-70.7%). For Muslim users, there is a noticeable gender difference with men consuming more illicit drugs than women [15]. In the western world, it is common for Muslim men and women to engage in smoking cannabis through their friends [15]. However, in Saudi Arabia, it has been found that women tend to engage in cannabis use through their families, and men initially use it through their friends [15]. Furthermore, women in Saudi Arabia who are single or divorced are more likely to consume cannabis than married women [15].

According to Bassiony, the most commonly used illicit substances in Saudi Arabia are amphetamines, heroin, alcohol, and cannabis [15]. Two decades ago in Saudi Arabia, the most prevalent illicit substance was heroin. However, a shift toward cannabis and amphetamines has been noted, with cannabis now being the most commonly used drug. The use of such illicit drugs can also be socially stigmatizing [15]. This is one of the most challenging community problems in Saudi Arabia, and there is yet to be any population-based studies undertaken on drug use and dependencies. Studies in this field have been limited to hospitals and treatment centers where most patients are males [15]. Further limitations include the fact that the studies usually encompass individuals who are already seeking help or patients admitted by their families or guardians. Therefore, the statistics do not represent the country as a whole but rather only a representative of individuals who are already undergoing treatment or enrolled in a program.

Risk factors

Bassiony reported that in Saudi Arabia, 68% of all substance abuse was initially due to peer pressure. Cannabis consumers similarly reported that their initial use of cannabis started with friends. Meanwhile, peer and group pressures are strongly linked to engagement with the drug [16]. This indicates that there is a high correlation between social relationships and drug use [17]. Furthermore, an astonishing 25% of cannabis users reported unhappiness in their marriage or social life [17]. A separate study in Saudi Arabia by Bassiony found that adolescents who abuse substances go through a four-step process in abusing illicit substances [17]. Data were collected using a questionnaire related to age and stages of progression of drug use. The study identified that the first step toward drug use is smoking tobacco, followed by the exploration of other drugs including cannabis, and further stages resulting in abuse of prescription medications [15]. Another study conducted in Tunisia found an even higher correlation between peer pressure and initial drug use [12]. Chekib et al. also found in their study that men were twice as likely as women to engage in substance abuse [12]. Gender itself is considered a risk factor when it comes to illicit substance use, especially in males [18]. However, in Egypt, it was found that female students are more likely to engage in drug use when compared to male students [19]. Academic failure can be related to cannabis use in two ways. On one hand, a student may use cannabis as a distraction from his/her low academic performance [12]. On the other hand, cannabis use can be directly correlated with lower academic performance, causing a reduction in academic motivation, possible cognitive ability impairment, and social disconnection [12]. Low economic status also seems to be a risk factor for substance abuse [12,20]. In Iran, a cross-sectional study conducted by Taremian et al. examined the risk and protective factors for substance use among university students. They reported that students with lower levels of religious belief were more likely to partake in substance use acts and events [20]. This demonstrates that high levels of religious belief can be considered a protective factor [11].

In a study conducted in the United Kingdom (UK), Williams et al. reported that the normalization of cannabis use within the Muslim youth community is increasing. Muslim youth in the UK tend to live in lower income housing with little access to higher level education and health services. These neighborhoods are also riddled with low employment. In comparison to other cultural groups, alcohol consumption was lower in these communities [18]. This process of normalization of cannabis is becoming accommodated in the everyday lives of Muslim youth. The availability of cannabis in these low-income neighborhoods has become so widespread that more youths are willing to try it, thereby making it tolerable for non-drug users over time [18].

Psychoactive experiences are increasingly a part of daily life for Muslim youth in the UK. Cannabis consumption now begins at younger ages in the UK. The accommodation of this consumption in their daily lives does not just affect social interactions but is also brought into one’s spiritual life [18]. Williams et al. demonstrated that there is a social stigma around cannabis use within the community. It was found that only half of the youths who were seeking help through a community-based program told their parents they were attending the programs [18]. This could mean that some youths are reluctant to get help or at least reluctant to seek help from their family because of the fear of the stigma associated with drug use. However, Williams et al.’s study was aimed at Muslim youth in the UK and does not take into account the global factor. It fails to consider major factors beyond the scope of the low-income communities where these youths reside. There is also no consideration of other outside forces or other forms of structural violence. Furthermore, there is a lack of understanding regarding the reasons for the attitude shift toward cannabis use. Interestingly, Muslim youths in the UK have begun to incorporate cannabis use even into their religious beliefs. For some, it has become normalized in their daily life. This suggests that cannabis use is impacting attitudes toward religion. Such an act is mainly associated with peers trying to fit into social situations and avoiding being stigmatized. In the UK, it was found that many migrant families live in low-income neighborhoods. As a result, social status plays a large role, along with religion, in shaping their attitudes toward cannabis use [21]. Delforterie et al. reported that teenagers born from immigrant parents are more likely to experience identity conflicts leading to stress and insecurity as well as an increase in the risk of deviant behavior [22]. There is pressure by the host culture on youth to assimilate for cultural acceptance. Here again, it is evident that peer groups have a large influence on youth engaging in cannabis use.

Prevention and treatment programs

There are currently no drug use prevention programs in Saudi Arabian schools [15]. Children and young adults are not educated on the harmful effects of cannabis or other illicit drugs. As these issues are not talked about openly in the community, the stigma of using illicit substances is still prevalent in Saudi society. While preventative programs have proved to be of great service to communities as a whole, users usually seek help late in Saudi Arabia [17]. When individuals receive help through treatment programs, it is not usually until the individual is admitted to a hospital for addiction treatment [17]. They are initially treated with detoxification, followed by behavior modification, and finally, rehabilitation. These programs include pharmacotherapy, psychotherapy, spiritual therapy, social services, education, and recreational activities [17]. The hospitals and rehabilitation centers utilize a biopsychosocial approach, treating addiction by incorporating different aspects of life into their treatment plans. They aim to treat the patient as a whole by viewing addiction as a mental, spiritual, and social sickness. This approach has great implications for specialists working with Muslim youth. Muslim youth are at risk of being stigmatized in their community, placing them at greater risk of addiction and the inability to seek help and education when necessary. Understanding the social dynamics of religion helps clinicians to provide a culturally sensitive environment in which the needs of Muslim youth can be addressed. Research regarding Muslim youth and cannabis use needs to focus on attitudes toward cannabis and other drug use. This will guide governments, policymakers, healthcare workers, and boards of education when managing Muslim youth policies, encouraging them to implement drug prevention programs in schools. A summary of the included studies and their outcomes are outlined in Table 1.

**Table 1:** Summary of included studies

Study ID	Participants	Purpose/Measure	Subgroup	Outcomes/Conclusions
Nahas (1982) [9]		This article gives a historic review of cannabis in Islamic history from the ninth to 18th centuries as well as a discussion on the social acceptance of cannabis in Islam.		Change is happening in the 20th century among the Islamic world with regard to science and cannabis.
Bradby and Williams (2006) [11]	824	Role of religion in abstinent behavior	British-born adolescents	Alcohol abstinence in Muslims
Bassiony (2008) [15]	101	Stages of progression in drug involvement among adolescents and adults	Adolescent (n = 10) Adult (n = 91)	Cannabis use (60.4%). Family history of substance abuse (21.8). Adolescents started using drugs at an earlier age compared to adults.
Kamarulzam and Saifuddeen (2009) [6]		This article discusses a key pillar of Islam, which is harm reduction, and how this principle must be used in reducing harms associated with substance abuse.		Harm reduction programs to reduce the effects and harms of substance abuse are considered a religious necessity and are completely concordant with the Islamic principles of the preservation and protection of the faith, life, intellect, progeny, and wealth.
Yassa et al. (2009) [8]	1,000	The present study was designed to determine the risk factors that lead to bango (cannabis) abuse among secondary school students and drivers in Assiut province in Egypt.	Adolescents and adults	The prevalence of cannabis use is 12%. Cigarette smoking is considered a gateway drug. Troubled familial relations led to higher rates of abuse (81%).
El-Sawi et al. (2010) [19]	457	Possible gender differences in the ways of first exposure to drugs, in their risks of abuse, and the pattern of drug dependence in Egypt	Adolescents and adults	Males started drug abuse earlier in age than females with a longer duration of addiction. Single males are more vulnerable to abuse than females. Drug abuse is more common in female students and male workers. Cannabis followed by opiates, then alcohol and analgesics are common in males, whereas in females, analgesics ranked first followed by anticholinergics, then cannabis. Peer pressure was the most common motivating factor for drug abuse in males.
Delforterie et al. (2014) [22]	771	Acculturation and affiliation in cannabis use in young and adult immigrants in the Netherlands	Immigrant adolescents and young adults aged 15–24 years.	Non-Western immigrant youngsters who speak the host culture's language at home are more likely to use cannabis than youngsters who speak their native language at home. The former group is more likely to affiliate with cannabis-using peers, which partly explains their increased risk of cannabis use. No relation between acculturation strategy and past-year cannabis use. Linguistic acculturation was positively related to cannabis use. Affiliation with cannabis-using peers partly mediated this relation.
Bassiony (2013) [17]		Substance use disorders in Saudi Arabia: a review article		There has been an increase in the use of cannabis and amphetamine and a decrease in the use of heroin and volatile substances. Peer pressure and psychosocial stress were major risk factors for substance abuse. Anxiety, depression, and hepatitis were the most common comorbid disorders among Saudi patients. Common comorbid disorders among Saudi patients.
Sattari et al. (2013) [10]		This article reviews Islamic history and substance abuse.		There is no evidence of tobacco use in the early days of Islam. There are no verses in the Quran prohibiting the use of cannabis and drugs, but given the multiple adverse effects of their use and multiple verses that forbid malevolence, many scholars prohibit the use of illicit drugs and cannabis.
Freeman et al. (2015) [4]	121	The objective was to determine whether the principal psychoactive ingredient of tetrahydrocannabinol causes paranoia and to use the drug as a probe to identify key cognitive mechanisms underlying paranoia		Tetrahydrocannabinol triggers paranoid thoughts in vulnerable individuals and triggers negative effects.
Chekib et al. (2016) [12]	556	To determine the prevalence of lifetime illicit substance use and its predictors in a college in Tunisia	College students	Illicit drug use was 5.6% (n = 31). Cannabis use was around 4.7% (n = 26). Proportions of male students and academic failure were significantly more important among illicit substance users than among non-users. The most influential factors on illicit substance use were alcohol use, tobacco use, and low socioeconomic level. There was a statistical significance between academic performance and drug use.
Telo et al. (2016) [14]	10,267	To determine the drug use prevalence in persons who admitted to the Probation Policlinic of Elazig Mental Health Hospital in East Turkey	Adolescent and adult	Cannabis is the most commonly abused drug in East Turkey with a prevalence of 32%. The prevalence of cannabis use was significantly higher in males. The prevalence of cannabis use was significantly higher in the 20-39 age group. The prevalence of cannabis use was significantly less in the 50-59 age group.
Volkow et al. (2016) [5]		Effects of cannabis use on human behavior, including cognition, motivation, and psychosis: a review		Current efforts to normalize cannabis use is being driven largely by a combination of grassroots activism, pharmacological ingenuity, and private profiteering, with a worrisome disregard for scientific evidence, gaps in our knowledge, or the possibility of unintended consequences. Vulnerable populations such as children, adolescents, the elderly, or individuals with other disorders may experience novel toxic effects.
Amin-Esmaeili et al. (2017) [16]	1,761	Prevalence of illicit substance use among students of Tehran University	Medical school students	Prevalence of last year's abuse of any illicit substance was 2.3% in 2006, 3.3% in 2007, 2.8% in 2008, and 1.1% in 2009. Substance abuse was significantly higher in male participants. Prevalence of use of Hasheesh (cannabis) was 1%-3%.
Williams et al. (2017) [18]	43	To examine the extent, frequency, and nature of substance use, and associated attitudes in ethnic groups in Britain (which includes individuals from India, Pakistan, and Bangladesh)	Adolescents	Extensive personal use of skunk cannabis in ethnic youth. Consumption of cannabis appeared to be accommodated into the daily lives of young ethnic groups and appears to be undergoing a process of normalization within these communities in Britain.
Almarhabi et al. (2018) [13]	101	Driving under the influence of an abused substance relative to age	Adolescent and adult	Amphetamines 56.4% (n = 57), alcohol 25.7% (n = 26), cannabis 24.8% (n = 25). Younger age at the time of the first substance abuse was associated with a higher probability of driving under the influence of an abused substance.

School-based prevention programs have the potential to be of great influence on vulnerable youth. These programs must be created in conjunction with religious beliefs. This will allow for Muslim youth to be educated culturally in an appropriate way by giving them an easy-to-grasp understanding of the dangers of cannabis and other forms of drug use. Furthermore, these programs will be able to offer students tools to resist drug offers from peers. Community-based programs may also provide discreet and anonymous interventions with families within the community. This would allow addicts and their families to receive the help and education they need without disgracing the individual or the family. These programs may involve education for the whole family, giving them tools to cope, manage, and build a stronger family, which in turn helps to strengthen the community as a whole.

## Conclusions

Current research on Muslim youth and cannabis use has consistently established that they are at a much greater risk than adults for substance use and abuse-causing behavioral and social problems. It is clear that there is a considerable lack of studies, research, and information regarding cannabis use in Muslim communities. This has a negative psychosocial effect on Muslim youth who are left uneducated about cannabis and other drug use.
